# The traits of the great calls in the juvenile and adolescent gibbon males *Nomascus gabriellae*

**DOI:** 10.1371/journal.pone.0173959

**Published:** 2017-03-15

**Authors:** Michal Hradec, Pavel Linhart, Luděk Bartoš, Petra Bolechová

**Affiliations:** 1 Department of Animal Science and Ethology, Czech University of Life Sciences Prague, Praha - Suchdol, Czech Republic; 2 Department of Ethology, Institute of Animal Science, Praha Uhříněves, Czech Republic; 3 Zoo Liberec, Liberec, Czech Republic; University of Lethbridge, CANADA

## Abstract

Knowledge about vocal ontogeny and vocal plasticity during ontogeny in primate species is central to understanding the evolution of human speech. Vocalizations in gibbons (*Hominoidea*) are very interesting and contain complex species- and sex-specific patterns. However, ontogeny of gibbon songs is little studied. Here, we document regular production and ontogenetic changes of female-specific “great call” in 4 immature (2 juvenile—c.a. 3 years old; and 2 adolescent—c.a. 5 years old) males of southern yellow-cheeked gibbon (*N*. *gabriellae*) over nine months. None of the males produced fully developed adult-like “great call” and little ontogenetic changes to “great calls” occurred. “Great calls” of sons were shorter, started higher and ended lower than those of their mothers. Regular production of twitter part of great call likely appears around 4^th^ year as it was observed in adolescent but not in juvenile males.

## Introduction

Gibbons are a relatively small, uniform group of territorial primates. Together with the other members of ape superfamily (*Hominoidea*), gibbons belong among closest extant human relatives. All gibbon species produce complex vocalisations, referred to as songs [1–3], making them an interesting group for comparative analyses on speech origins [4]. Gibbon males and females usually sing in duet [3]. The vocal repertoire of the gibbons is sex-specific. The degree of repertoire sharing between males and females depends on species with one exception. A “great call” has been originally reported as specifically produced by females only in adult gibbons [3]. More recently it has been documented, however, that immature *Hylobates* males can produce “great calls” [5]. It remains unclear whether immature *Hylobates* males produce the “great calls” regularly as a part of normal vocal development [5]. On the other hand, regular production of “great calls” was mentioned without more detailed investigation in northern white-cheeked gibbon (*Nomascus leucogenys)* immature males [6, 7] and we observed regular production in southern yellow-cheeked gibbons as well (MH pers. observation). Thus, southern yellow-cheeked gibbon and likely other gibbon species too offer the opportunity to investigate how sex-specific roles emerge during the duet ontogeny and to compare the ontogeny of the same vocalization in both sexes.

**Fig 1:**
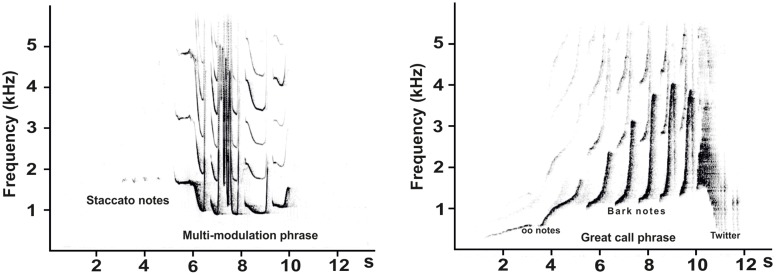
Typical male’s (left) and female’s (right) spectrogram showing the vocalization of southern yellow-chekeed gibbons.

In southern yellow-cheeked gibbon, adult males produce sound structure that consists of a “staccato” note and a “multi-modulation” phrase (coda phrase) (Fig 1). “Staccato” notes are very subtle and produced in short, irregular series. “Multi-modulation” phrases belong to dominant acoustic structures [2, 3, 8]. A female-specific vocalization, the “great call”, has typically 5–13 notes that further split into”oo” notes, “bark” notes and a”twitter” part (Fig 1)[2, 3, 8–10].

The aim of this study was to describe vocal development of juvenile and adolescent males (immature males) of southern yellow-cheeked gibbon in captivity. In southern yellow-cheeked gibbon female developed adult-like “great call” before she was three years old [9]. "Great call" in immature female had almost the same number of syllables with a regular presence twitter as her mother. In addition, the delay of the daughter’s “great call” following her mother’s “great call (“delay”) decreased with age of the daughter. We predicted, that “great call” in immature males (*i*) will have the same number of syllables, (*ii*) it will contain the “twitter” and (*iii*) delay will decrease with age of immature males.

## Materials and methods

### Ethic statement

This study was authorized by managements of Olomouc and Jihlava Zoos. Both zoological institutions have rigorous standards for animal welfare and are accredited by the EAZA (European Association of Zoos and Aquaria) and UCSZOO (Union of Czech and Slovak ZOOS). Our study was non-invasive and fully complied with the legal requirements of the Czech Republic (Czech National Council Act No. 246/1992 Coll. the protection of animals against cruelty, amended by Act No. 162/1993 Coll.). The record was done from the outside of the gibbon´s facility.

### Subjects

Our research was conducted in two Czech zoological parks that house southern yellow-cheeked gibbons (see overview in Table 1). According to Reichard [11], immature males were divided into the categories of juvenile and adolescent, because the difference between them was 12 months (juvenile male from 2yr 3m to 3yr 7m, adolescent from 4yr 7m to 6yr).

**Table 1:** Information about subjects.

General information								
**ZOO**	**Olomouc**				**Jihlava**			
**Group**	1				2		3	
**Name**	Benjamin		Silvestr		Jihlava 1		Jihlava 2	
**Date of birth**	12 December 2008		31 December 2011		14 October 2009		2 April 2011	
**Age of the males during the study**	5yr 3m – 6yr		2yr 3m – 2yr 12m		4yr 7m – 5yr 1m		3yr 1m – 3yr 7m	
	Session	Date	Session	Date	Session	Date	Session	Date
**Recording dates and number of session (all recordings were taken in 2014)**	**1**	11–14 March	**1**	11–14 March	**1**	27–29 May	**1**	27–29 May
	**2**	8–9 April	**2**	8–9 April				
	**3**	30 April–2 May	**3**	30 April–2 May	**2**	6–7 November		
	**4**	9–11 October	**4**	9–11 October			**2**	6–7 November
	**5**	12–14 November	**5**	12–14 November	**3**	18–19 November		
	**6**	3 and 10 December	**6**	3 and 10 December				
**Number of recorded “great calls” mother**	132		132		45		35	
**Number of recorded “great calls” juvenile and adolescent males in duet with mother**	120		120		41		27	

We studied vocalisation of 2 juvenile and 2 adolescent males (2y 3m – 5y 3m, age at the beginning of the study) from three groups. Since juvenile females and males do not differ in coat colour, sex of juvenile and adolescent males was determined during routine veterinary examinations. All these individuals were housed with their family group consisting of an adult gibbon pair and their progeny. The only exception was an adolescent male from Jihlava 1. This group consisted of the studied adolescent male, his mother and his older brother. Jihlava and Olomouc zoos housed two groups of *Nomascus* gibbons being in visual and auditory contact.

### Recording and acoustic analysis

Vocalization were collected from March to May 2014 and from October to December 2014. Each zoo was visited 2 to 6 times (1 zoo visit = session). Spontaneous vocalizations were recorded from 5:00 to 12:30 a.m. Gibbons were recorded within a distance of 2 to 15 metres.

We recorded songs using the M-Audio Micro Track II recorder with Rode NTG-2semi-directional microphone. Sounds were recorded in mono with 16-bit resolution and 96 kHz sampling rate. All the recorded songs were saved as wav files. Sampling frequency was reduced from 96 kHz to 12 kHz for further analyses. Acoustic analysis was performed using Avisoft SASLab pro version 5.2 (Avisoft Bioacoustics, Berlin, Germany). Spectrograms were generated with following settings (FFT length = 1024, frequency resolution = 12 Hz, temporal resolution = 21,3ms, overlap = 75%, window type = Hamming). Duetting was video recorded using the Nikon Coolpix L330 camcorder with 30x zoom (S1 Video).

**Fig 2:**
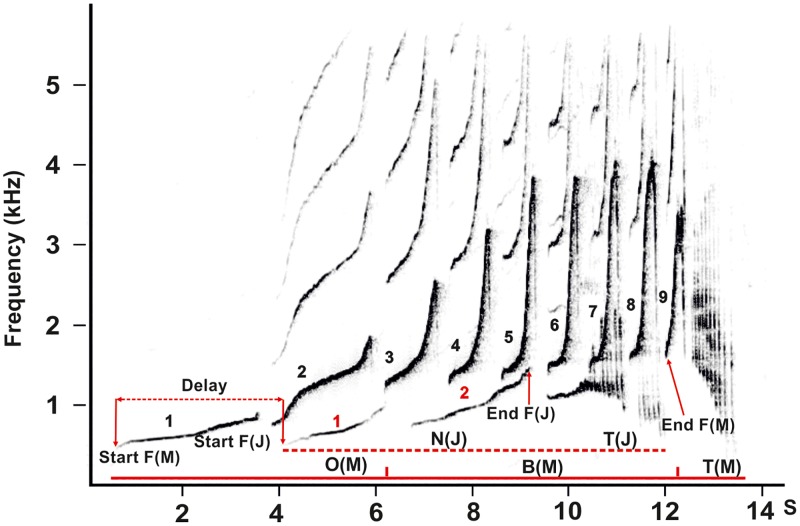
Representative spectrogram showing the parameters measured: Start F (frequency at call onset), End F (frequency at call offset), Delay; Number of syllables (black number = syllable mother, red number = syllable juvenile male) and song organization of mother’s (M) and juvenile’s (J) “great calls”. Dashed line represents notes of juvenile’s “great call”: N(J)–note juvenile, T(J)–“twitter” juvenile; solid line represents notes of mother’s “great call”: O–“oo” notes, B–“bark” notes, T–“twitter”.

Due to the overlap of two or even three “great calls” (mother singing simultaneously with offspring), it was difficult to apply any detail acoustic measurements. However, basic spectro-temporal measurements could be reliably distinguished (Fig 2). To characterize “great calls” we counted the number of syllables preceding a “twitter” (‘SylN’), noted presence of the “twitter” (‘Twitter’), to characterise temporal synchrony between adult female and males we computed difference in onset time of their great calls (‘Delay’). These measurements correspond to those used to study ontogeny of southern yellow-cheeked gibbon “great calls” by juvenile female [9]. In addition, to characterise basic spectral properties of the “great calls” we measured an onset frequency (‘Start F’, beginning of the first syllable) and the offset frequency (‘End F’, at the beginning of the last syllable preceding the twitter).

### Statistical analyses

All data were analysed with the aid of SAS System version 9.4 (SAS Institute Inc.). As a Generalized linear mixed model (GLMM) for modelling the probability that an adolescent gibbon male will produce a “twitter” in a duet with his mother we applied a PROC GLIMMIX for binary distribution with a “twitter”as a dependent variable. The link function was logit and all error terms were binomial in the GLMM. A delay between the mother and an immature male (“Delay”) as a dependent variable was analysed with PROC MIXED. The goodness of fit of each model (homoscedasticity, normality of errors and independence) was checked by visual inspection of residuals using plots = pearsonpanel (PROC GLIMMIX) and plots = all (PROC MIXED) and testing residuals for normality by Kolmogorov-Smirnov test. Any factors which did not add to significance (P > 0.05) were dropped from the model and will not be mentioned any further. Where appropriate we tested interaction terms. In both GLMM procedures, the fixed effects were log-transformed (to improve normality of residuals and to reduce skewness) age of the male (37 to 62 months) and number of syllables in a son ´s call (2 to 5), and categorical effects Zoo (Jihlava or Olomouc), and an ID of the mother (3 levels). The significance of fixed effect in the GLMM was assessed by the F-test. Both analyses were designed with an ID of the male as a random effect. Associations between the dependent variable and fixed effects were estimated by fitting a random coefficient model using PROC MIXED as described by Tao et al. [12].

## Results

### Great calls in immature *N*. *gabriellae* males

We document here that juvenile and adolescent southern yellow-cheeked gibbon males in captivity regularly produce a "great call" in a duet with their mother (S1 Video). “Great calls” by juvenile and adolescent males were frequent observed during each session (see overview in Table 1). All males already produced “great calls” since the first recording session.

### Changes in great call parameters during ontogeny

**Fig 3:**
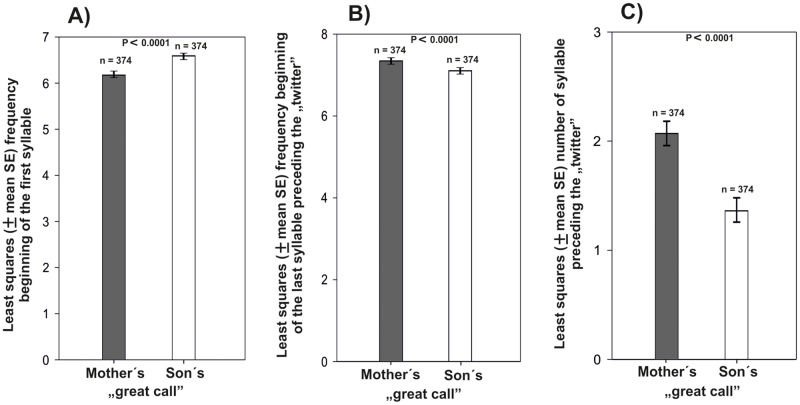
Differences in characteristics of adult female´s and their son´s “great calls” (least squares mean ± SE). A) start frequency of the first syllable, B) start frequency of the last syllable, and C) number of syllables preceding the”twitter”.

Neither “great calls” of juvenile nor those of adolescent males fully resembled “great calls” of adult females. In both ZOOs, immature males (adolescents and juveniles) had higher first syllable, lower last syllable (F_(2, 181)_ = 23.24, P < 0.0001, Figs 2, 3A and 3B) and lower number of syllables than adult females (F_(2, 181)_ = 23.24, P < 0.0001, Fig 3C). Also, all immature males began singing with a considerable delay after the mother started her singing.

**Fig 4:**
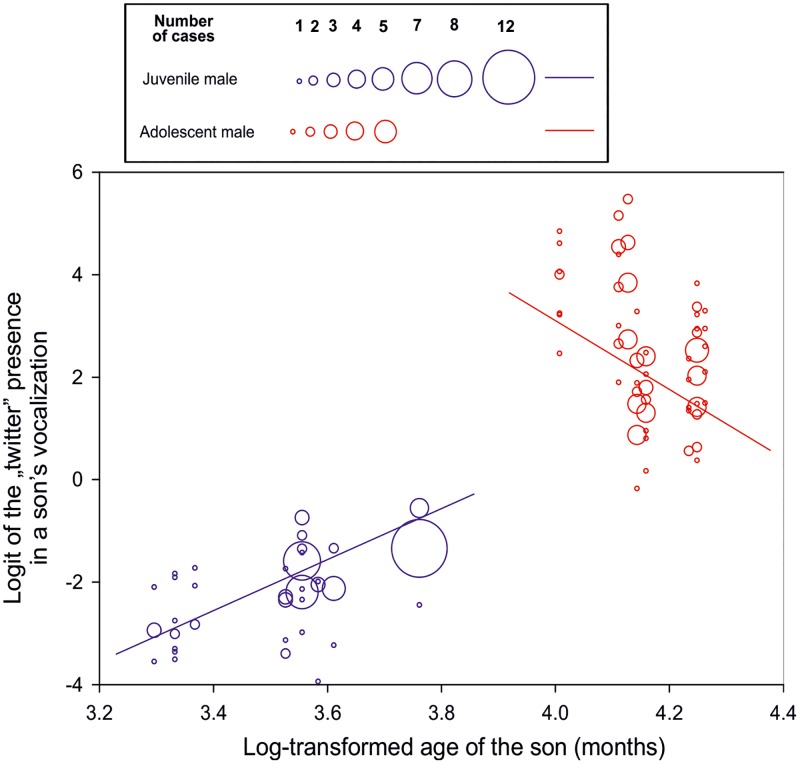
Logit of a “twitter” occurrence in the “great call” of the son plotted against log-transformed age of the juvenile or adolescent son.

**Fig 5:**
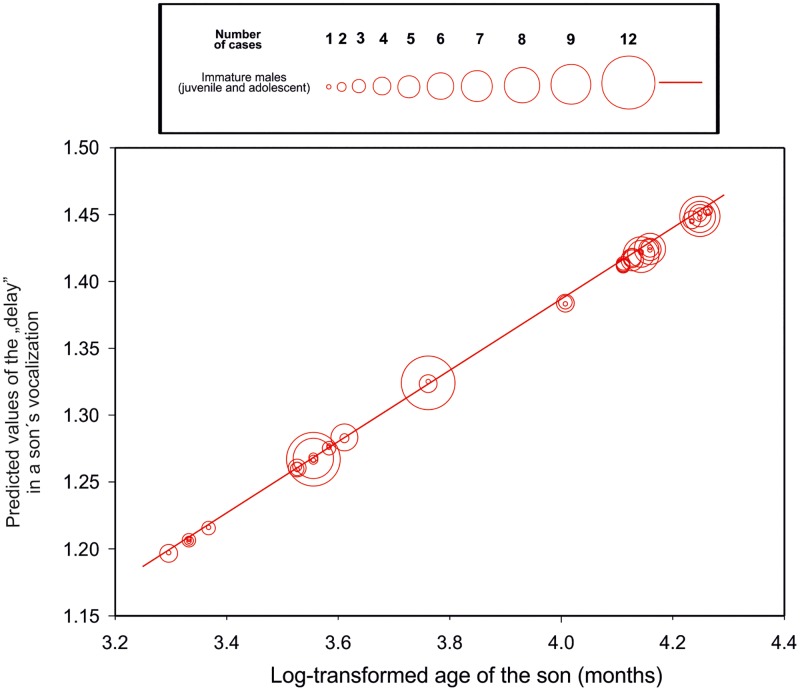
Predicted values of the delay in temporal synchrony of great calls emitted by an adult female and her son plotted against log-transformed age of the son.

Adolescent males produced consistently across the two ZOOs higher proportion of the “twitter” than juvenile males. Probability of a “twitter” presence increased with increasing age of the male (F_(2, 181)_ = 23.24, P < 0.0001, Fig 4). The “Delay” was decreasing with increasing age of the immature males (F_(1, 181)_ = 41642.8, P< 0.0001, Fig 5).

## Discussion

Our study represents first contribution that systematically describes the ontogeny of female-specific “great calls” in immature males. In this study, we documented that juvenile and adolescent males of southern yellow-cheeked gibbon produced regularly female-specific vocalisation, “great calls” in a duet with their mothers. Nevertheless, parameters of “great calls” produced by juvenile and adolescent southern yellow-cheeked gibbon males did not converged to complexity of the adult female “great calls”, with the exception of “twitter” occurrence. “Twitter” was almost absent in juvenile males but common in adolescent males.

### Great call as a common part of *N*. *gabriellae* male vocal ontogeny

Ontogeny of gibbon duets has received little attention and the mechanisms how the males and females get into their sex-specific duet roles remain unclear. Few studies mention that immature males produce female specific vocalizations in the *Hylobates* and *Nomascus* genera, whether in the wild or in captivity [3, 5, 7, 13]. Deputte [6] and Hradec [7] briefly mentions regular production of “great calls” in juvenile males of northern white-cheeked gibbon. Even in more detailed study, Koda et al. [5] reported immature agile gibbon (*Hylobates agilis)* and lar gibbon (*Hylobates lar*) males to give female “great calls” sporadically, leaving doubts whether this behaviour is common or not. In our study, we regularly observed immature southern yellow-cheeked gibbon males to emit female “great calls” and, therefore, we suggest that production of “great calls” is an integral developmental phase of vocal ontogeny at least in southern yellow-cheeked gibbon.

### Ontogeny of male great call in *N*. *gabriellae*

“Great calls” appear apparently before males reached 2 years of age as female-specific “great calls” were already present in all the males at the beginning of the study (Silvestr, 2y 3m). in immature males (juvenile and adolescent) “great calls” are much simpler than in adult females and showed little capacity to develop in full “great calls” in our study Also, spectral parameters of “great call” in immature males were different from mother´s great call (prediction *i*). Spectro-temporal parameters of juvenile (c.a. 3 years) and adolescent (c.a. 5 years) males were similar. Regular production of “Twitter” develops between c.a. 3–5 years. We have no data available when immature males begin to sing “great calls” and how their first “great calls” look like. This is still an open field for further investigations.

### Comparison of great call ontogeny in males and females

Contrary to what was found in developing females, our results show that female-specific “great call” in immature males is does not mature into the fully developed, adult-like “great call”. Ontogeny of “great calls” emitted by immature males and females seem to be different. Earlier study showed that a young female developed the “great call” much like that of her mother at about 32 months of age [9]. Daughter’s great call reached the same number of elements as her mother’s, while sons emitted much lower number of elements per “great call” (prediction *i* not supported). Although, we used slightly different method for “twitter” reporting than previous study, it is apparent that “twitter” is regularly present later in males than in females. It was regularly used by 32 months old immature female. On the other hand, “twitter” was still scarce in our juvenile males (> 32 months old) and was regular only in adolescent males (prediction *ii*). Further, daughter sang in greater synchrony with mother and onset of her songs were bellow 1s after the onset of mother’s “great call”, while sons start to sing with app. 3s delay after mother. However, the length of the parameter “delay” in son´s were influenced by their age (prediction *iii*). The earlier study did not evaluate any spectral features of “great calls” but it seems from available spectrograms that daughter matched the pattern of the mother’s “great call” more closely than sons in our study. Matching the specific pattern of mother’s “great call” observed between agile gibbon mothers and their sub-adult daughters [5] might not be possible or necessary for immature males.

### Why juvenile and adolescent males produce female-specific vocalizations?

In gibbons and some other primates, group vocalization plays an important role in maintaining group cohesion, pair bond and territoriality [14–17]. The genus *Nomascus* has most marked sex-specificity vocalization in male and female of all gibbons. Additionally, there is no overlap vocal pattern between them [3]. This could facilitate vocal interaction between mother and immature individuals. However, it seems that vocal interaction between mother and her offspring contribute to the development of vocalization and behaviour. The acoustic convergence between daughter and her mother led to development of female full-fledged pattern of the „great call”[9, 18] and acquisition of social independence followed with emigration of immature females from the family group. Our data suggest a similar role of a mother in the vocal development of her immature son. According to Burns et al. [19,20] immature males start maturation around the 5 year of age, which continues until at least the 7 year of age. At this stage the immature males tend to remain closer to the mother, because in this period the father increases aggression against them. Producing the “great call” in juvenile and adolescent males could fulfil an important task of strengthening family ties and at the same time passing the information about the immature status of the singing male to the father. Gibbons are territorial and bouts of singing from other males trigger aggressive behaviour [15, 17]. Therefore, using female specific vocalizations is a way how juvenile and adolescent males might be tolerated by the adult male to participate in group vocalizations and practice vocal abilities.

The more detailed studies are needed to reveal patterns and mechanisms of vocal ontogeny in gibbons. The detailed knowledge of gibbon song vocal ontogeny could help us better understand the evolution of singing behaviour in this taxon. Also, studying vocal ontogeny of gibbons rises important questions to what extent the vocal behaviour in this group is genetically determined or whether it could be influenced by learning.

## Supporting information

S1 Video (MP4)
